# Complete Genome Sequence of an Indian Field Isolate of Classical Swine Fever Virus Belonging to Subgenotype 1.1

**DOI:** 10.1128/genomeA.00886-14

**Published:** 2014-10-02

**Authors:** Aman Kamboj, Chhabi L. Patel, V. K. Chaturvedi, Mohini Saini, Praveen K. Gupta

**Affiliations:** a Division of Veterinary Biotechnology, Indian Veterinary Research Institute, Izatnagar, Uttar Pradesh, India; b Division of Biological Products, Indian Veterinary Research Institute, Izatnagar, Uttar Pradesh, India; c Center for Wildlife, Indian Veterinary Research Institute, Izatnagar, Uttar Pradesh, India

## Abstract

We report the complete genome sequence of an Indian field isolate of classical swine fever virus (CSFV) belonging to predominant subgenotype 1.1 prevalent in India. This report will help in understanding the molecular diversity of CSFV strains circulating worldwide and to select and develop a suitable vaccine candidate for classical swine fever (CSF) control in India.

## GENOME ANNOUNCEMENT

Classical swine fever (CSF) is a highly contagious, fatal disease of pigs and wild boars caused by the CSF virus (CSFV), a pestivirus belonging to the family *Flaviviridae*. This is a World Organisation for Animal Health or Office International des Epizooties (OIE)-notifiable disease prevalent worldwide, including most parts of India. The only serotype of CSFV isolates reported worldwide has been placed into three genotypes (1–3). Each of the first 2 genotypes are divided into 3 subgenotypes (1.1, 1.2, 1.3, 2.1, 2.2, and 2.3) and the third genotype is divided into 4 subgenotypes (3.1, 3.2, 3.3, and 3.4) (1). Among the subgenotypes (1.1, 2.1, 2.2) prevalent in India, subgenotype 1.1 is predominant (2, 3).

The CSFV genome is a linear positive-sense single-stranded RNA of approximately 12.3 kbp in length. This contains a single open reading frame (ORF) of 11.7 kbp flanked by 5′ and 3′ non-translated regions (NTRs) (1). The ORF is translated into a single polypeptide of about 3,900 amino acids which is co- and post-translationally processed into 4 structural proteins *viz*., C, Erns, E1, and E2 and 8 nonstructural proteins *viz*., Npro, p7, NS-2, 3, 4A, 4B, 5A, and 5B (4).

In this study, we report complete genome sequence of a CSFV isolate (CSFV/IVRI/VB-131) isolated from spleen of a suspected case from a backyard pig. The complete genome was amplified using reverse transcription-PCR (RT-PCR) in six overlapping fragments. Total RNA was isolated using Trizol reagent (Invitrogen, USA), and cDNA was synthesized with AccuScript High Fidelity reverse transcriptase (Agilent, USA) using CSFV-specific oligonucleotide primers. The overlapping genome fragments were amplified from cDNA using Q5 Hot Start High-Fidelity 2× Master Mix (New England BioLabs, USA) and cloned into pJET1.2/blunt cloning vector (Thermo Scientific, USA). The recombinant plasmids were sequenced using primer walking strategy with an ABIPRISM 3730xl DNA Analyzer. The generated sequences were annotated and assembled using SeqMan Pro 7.1 (DNASTAR, Lasergene, USA) to form a single contig. The consensus sequence of CSFV/IVRI/VB-131 covered the entire length of the 12,300 nucleotides (nt), including a 373-nt 5′ NTR, an 11,697-nt ORF encoding a 3,898-amino acid long polyprotein and a 230-nt 3′ NTR. The genome organization of CSFV/IVRI/VB-131 was similar to other CSFV strains from different parts of the world.

Phylogenetic analysis with MEGA6 (5) using the other CSFV complete genome sequences (*n* = 60) available in GenBank, the CSFV/IVRI/VB-131 clustered to a common node with strains belonging to subgenotype 1.1 with percent identity from 93.0 to 97.6%. The percent identity was 93.7 to 94.3%, 84.4 to 85.3%, 83.5 to 86.2%, and 85.0 to 85.8% with CSFV strains belonging to subgenotype 1.2, 2.1, 2.2, and 2.3, respectively.

Although CSFV subgenotype 1.1 is predominant in India, this is the first report on isolation and complete genome sequencing of a CSFV field isolate belonging to subgenotype 1.1 from India. The complete genome sequence information of the CSFV/IVRI/VB-131 isolate in the present study will help in understanding the molecular diversity of CSFV strains circulating worldwide and to select and develop an effective vaccine candidate for CSF control in India.

### Nucleotide sequence accession number.

The complete genome sequence of CSFV/IVRI/VB-131 has been submitted to GenBank under the accession no. KM262189.

## References

[1] Paton DJ McGoldrick A Greiser-Wilke I Parchariyanon S Song JY Liou PP Stadejek T Lowings JP Björklund H Belák S 2000 Genetic typing of classical swine fever virus. Vet. Microbiol. 73:137–157. 10.1016/S0378-1135(00)00141-3 10785324

[2] Desai GS Sharma A Kataria RS Barman NN Tiwari AK 2010 5′-UTR-based phylogenetic analysis of classical swine fever virus isolates from India. Acta Virol. 54:79–82. 10.4149/av_2010_01_79 20201618

[3] Kumar R Rajak KK Chandra T Thapliyal A Muthuchelvan D Sudhakar SB Sharma K Saxena A Raut SD Singh VK Ahmad Z Kumar A Chaudhary D Singh RK Pandey AB 2014 Whole-genome sequence of a classical swine fever virus isolated from the Uttarakhand state of India. Genome Announc. 2(3):e00371-14. 10.1128/genomeA.00371-14 24812219 PMC4014687

[4] Falgout B Markoff L 1995 Evidence that flavivirus NS1-NS2A cleavage is mediated by a membrane-bound host protease in the endoplasmic reticulum. J. Virol. 69:7232–7243 7474145 10.1128/jvi.69.11.7232-7243.1995 PMC189645

[5] Tamura K Stecher G Peterson D Filipski A Kumar S 2013 MEGA6: molecular evolutionary genetics analysis version 6.0. Mol. Biol. Evol. 30:2725–2729. 10.1093/molbev/mst197 24132122 PMC3840312

